# Systematic development of a self-regulation weight-management intervention for overweight adults

**DOI:** 10.1186/1471-2458-10-649

**Published:** 2010-10-27

**Authors:** Lenneke van Genugten, Pepijn van Empelen, Ilse Flink, Anke Oenema

**Affiliations:** 1 Department of Public Health, Erasmus Medical Center, PO Box 2040, 3000 CA Rotterdam, The Netherlands

## Abstract

**Background:**

This paper describes the systematic development of an intervention for the prevention of obesity among overweight adults. Its development was guided by the six steps of Intervention Mapping (IM), in which the establishment of program needs, objectives and methods is followed by development of the intervention and an implementation and evaluation plan.

**Methods:**

Weight gain prevention can be achieved by making small changes in dietary intake (DI) or physical activity (PA). The intervention objectives, derived from self-regulation theory, were to establish goal-oriented behaviour. They were translated into a computer-tailored Internet-delivered intervention consisting of four modules. The intervention includes strategies to target the main determinants of self-regulation, such as feedback and action planning.

The first module is intended to ensure adults' commitment to preventing weight gain, choosing behaviour change and action initiation. The second and third modules are intended to evaluate behaviour change, and to adapt action and coping plans. The fourth module is intended to maintain self-regulation of body weight without use of the program.

The intervention is being evaluated for its efficacy in an RCT, whose protocol is described in this paper. Primary outcomes are weight, waist circumference and skin-fold thickness. Other outcomes are DI, PA, cognitive mediators and self-regulation skills.

**Discussion:**

The IM protocol helped us integrating insights from various theories. The performance objectives and methods were guided by self-regulation theory but empirical evidence with regard to the effectiveness of theoretical methods was limited. Sometimes, feasibility issues made it necessary to deviate from the original, theory-based plans. With this paper, we provide transparency with regard to intervention development and evaluation.

**Trial registration:**

NTR1862

## Background

Because of its association with several negative health outcomes [1-3] and increased health care costs [4], the high and increasing prevalence of obesity is a major public health concern. In 2005 there were about 1.1 billion obese adults worldwide [5]. Recent Dutch data (2005-2008) showed that 11% of adult males and 12% of adult females were obese [6]. Although behavioural treatment of obesity has improved greatly over the past 20 years, reductions in weight are rarely maintained among obese people [7]. Given these poor results, the prevention of obesity has been postulated as a promising strategy for fighting the obesity epidemic [6]. This includes weight maintenance or modest weight loss, from here on referred to as weight-management [8], which can be achieved by restoring the balance between energy intake and energy expenditure.

Overweight adults (BMI 25-30 kg/m^2^) are an especially important group to target with obesity prevention interventions: not only are they most at risk of becoming obese, they also comprise a large group. In the Netherlands, more than 28% of women and 41% of men were overweight in 2005-2006 [9].

There is a lack of well designed, theory and evidence-based interventions that focus on weight-management among adults being overweight. Such an intervention should be able to reach a large group of overweight adults and has to take the large differences between people in their behaviours, preferences and capabilities into account. Therefore, individualized intervention approaches are needed to successfully modify weight-related behaviours [10]. We initiated a project to develop and evaluate such an intervention.

In this paper we describe the results of the planned development of the intervention and the evaluation protocol. The development was guided by the Intervention Mapping (IM) protocol to ensure that the intervention was grounded in theory and evidence and to maximise the likelihood of effectiveness [11]. The IM protocol distinguishes six steps in the (iterative) process of developing an intervention, implementation and evaluation plan. The first step is the needs assessment, which results in a description of the health problem addressed, its behavioural causes and the intervention goal. The second step is aimed at stating specific change objectives, the most detailed and proximal goals that will be addressed in the intervention. In the third step, theoretical methods and practical strategies that are suitable to reach the change objectives are identified. In step four the actual program is developed and pre-tested. Step five and six involve the development of an implementation and evaluation plan. In the present paper we will specifically focus on the results of steps two to four. By detailing these steps specifically we comply to recent calls for specific descriptions of interventions that will increase transparency of intervention content and improve the options for replication [12,13].

## Methods and design

The results of each step of the development process are described below.

### Step 1 Needs assessment

As determined by the needs assessment, which is briefly summarised in the introduction section, the overall goal of the intervention was to prevent weight gain in overweight adults. Weight gain prevention (WGP) does not require dieting, but can be achieved by making small, but sustained changes of about 100 kcal a day in energy intake and/or energy expenditure. Dietary intake (DI) can be reduced by making changes in food categories that contribute most to excess energy intake and obesity, namely alcoholic drinks, sugar sweetened drinks and juices, and energy-dense foods (high in fat and/or sugar) [14]. Physical activity (PA) can be increased by activities in the various PA sub-domains, namely active transport and activities at work, during leisure time (walking or cycling), and sports [14]. An increase in PA of about 20 minutes a day is equivalent to a 100 kcal increase in energy expenditure. Preferably, the activities should be of moderate to vigorous intensity. Thus, the overall goal of the intervention can be achieved by making small (at least 100 kcal per day) and sustained changes in DI and/or PA.

### Step 2 Matrices of change objectives

The overall intervention goals cannot be achieved directly, but only through targeting specific behavioural actions that are needed to achieve them (e.g. reduce intake of high-energy snacks). The definition of these most specific program goals occurs in two steps. In the first step we defined performance objectives (POs), in the second step we defined change objectives (COs). POs specify the behavioural actions that the target audience has to perform in order to successfully change behaviour (e.g. reduce the intake of sugar sweetened drinks by 1 glass per day).

#### Performance objectives

Because weight gain prevention requires long-term self-management skills to regulate and adapt behaviour to changing circumstances, self-regulation models were used to guide the definition of the POs. Self-regulation models (e.g [15,16]) describe sub-behaviours that are necessary to establish and maintain changes in complex behaviours. Five different POs were defined and involved establishing, setting, planning, striving, revising and maintaining a goal:

(1) People decide to prevent weight gain (goal establishment).

(2) People choose at least one small change in DI or PA (goal setting).

(3) People prepare strategies to establish how they will make their chosen behaviour change (planning).

(4) People change their DI or PA (goal striving).

(5) People evaluate the success of the behaviour change and its effect on body weight (goal monitoring, attainment, revision and persistence decisions).

(a) if successful, they may maintain or adapt their goal,

(b) If unsuccessful, they go back to previous stages and revise their strategies for them (#5)

(c) if unsuccessful, they may also choose a new behavioural goal (#2)

These objectives provide a sequence of actions but are also circular in nature, meaning that recycling to previous steps in the self-regulation process is possible.

#### Selecting determinants

Translating the POs into more specific change objectives requires a thorough analysis and selection of the most important and changeable determinants of each PO (and thus phase in self-regulation). Our analysis of determinants was based on a review of empirical determinant studies and relevant motivational and volitional theories such as self-regulation theory [15,16], the Theory of Planned Behaviour [17], the Precaution Adoption Process Model [18], implementation intentions and goal setting [19], and Relapse Prevention Theory [20]. It was also based on the results of focus group interviews (FGI) held with the target group.

The study of determinants showed that awareness and risk perception, knowledge [21,22], attitude and perceived behavioural control [17] are important determinants. Table 1 provides a selection of the most important determinants for each PO.

**Table 1:** Performance objectives for preventing weight gain, with a selection of determinants per performance objective

Performance Objective	Theory	Determinants
1. People decide to prevent weight gain.	PAPM [22]	- Awareness. - Knowledge - Risk-perception [23]
	
	TPB [17]	- Attitude - Perceived Behavioural Control

2. People choose at least one small change in DI or PA (goal setting).	PAPM	- Awareness
	
	TPB	- Attitude/preferences [33] - Subjective norm (FGI), [34,35] - Goal-efficacy (FGI)
	
	SDT [36]	- Goal commitment.

3. People prepare strategies to establish how they will make their chosen behaviour change	HAPA [37]	- Action self-efficacy [38-40] - Awareness of cues to action
	
	TPB	- Social influence [41]

4. People change their DI or PA (goal striving).	HAPA	- Action self-efficacy [42] - Coping self-efficacy (FGI)
	
	RPT [20]	- Awareness of barriers/high risk situations - Coping self-efficacy [20]
	
	SRT [16]	- Awareness of standards (their self-chosen change) - Monitoring (self-regulation effort)

5. People evaluate their behaviour change and its effect on body weight.	SRT (also for 5a, 5b and 5c)	- Awareness of personal weight standards - Skills

a. if successful, they may maintain or adapt their goal (towards a higher goal).	HAPA	- Task self-efficacy
	
	SDT	Commitment

b. If unsuccessful, they go back to previous stages (#5) and revise their strategies for them	RPT	- Recovery self-efficacy [43]
	
	SDT	- Commitment
	
	TPB	- Attitude [20,44,45]. - Awareness
		
c. if unsuccessful, they may also choose a new behavioural goal (#2)		

***FGI **= information derived from Focus Group Interviews, **GST **= Goal Setting Theory, **HAPA **= Health Action Process Approach, **PAPM **= Precaution Adoption Process Model, **RPT **= Relapse Prevention Theory, **TPB **= Theory of Planned Behaviour, **SDT **= Self determination Theory, **SRT **- Self-Regulation Theory*.

#### Writing change objectives

In the last phase of step 2 we defined the change objectives (COs). This is an important step, since these define what the target audience has to learn or change in order to be able to perform the specific behaviours and are therefore the most direct targets of the intervention. A matrix of change objectives, was developed by crossing the behavioural determinants and POs. In total, almost 200 COs were defined to design the program, a selection of which is presented in the first column of table 2.

**Table 2:** Selected change objectives, theoretical methods and practical strategies

Change objectives The participant:(determinant)	Theoretical Methods (all tailored)	Parameters for use	Practical strategy
**PO 1. People decide to prevent weight gain**.			

1.1 Acknowledges personal weight changes in past (awareness) Acknowledges risk of possible future weight gain and its health consequences (risk perception)	Provide feedback using images. Personalised scenario based risk information [23].	Familiar physical or verbal images as analogies to a less familiar process. Plausible scenario with a cause and outcome; imagery. Presented as individual and undeniable.	Weight development over past 5 years is shown in a graph after answering questions about weight history. Trend for weight development is predicted (e.g. weight gain when no action is undertaken) and compared to the intervention goal: lifelong weight-gain prevention.

1.2 Can explain what the energy balance is, its relation to body weight and small changes in DI and PA (knowledge).	Provide information about behaviour-health link. [11].	Message is relevant and not too discrepant from target's group experience.	Short pieces of factual information about the energy balance, bodyweight, and small changes. Illustrations are added to clarify the text.

1.3 Has stronger positive feelings towards WGP than negative (attitude).	Prompt review of current behavioural goals/perspective [46] Anticipated regret [47].	Initiation from the perspective of the learner. Neutrality of original attitude.	Users fill out advantages and disadvantages of WGP, which results in a decisional balance. They are asked to (re)consider their advantages and disadvantages and relative importance and decide whether they are willing to prevent weight gain. Those who do not yet decide for WGP are asked to consider the long-term consequences of weight-gain prevention and 'no action', and can then re-consider their choice.

1.4 Says to be able to prevent weight gain. (self-efficacy)	Provide general encouragement by modelling	Attention, remembrance, skills, reinforcement; credible source, method and channel.	People are asked if they think they can prevent weight gain. If not, some peers tell their positive experiences with WGP (testimonials).

**PO 2. People choose at least one small change in DI or PA**.			

2.1 Is able to describe personal DI and PA (awareness)	Personal feedback on behaviour [28,48-51]	Feedback that is individual, follows the desired behaviour closely in time.	They fill out detailed questions on DI and PA. Individual feedback on DI and PA is given, and areas for improvement are indicated. (Oenema, Tan et al. 2005; Oenema, Brug et al. 2008)

2.2 Chooses a change that they feel positive and self-efficacious about (goal commitment + action efficacy).	Prompt intention formation by belief selection [11]	Requires investigation of the current beliefs of the individual before choosing the belief on which to intervene.	The program allows users to choose one change from a personal list. People are asked to pick a change that they think they *can *change and would *enjoy*.

2.3 States a clear goal	Guided goal setting [52].	Commitment to the goal; goals that are difficult but available within the individuals practice of coping response.	People set a clear goal, guided by questions in a graphic organiser, such as the size of the change the would like to make./Their answers are presented as their personal goal.

**PO 3. People prepare strategies to establish how they will make their chosen behaviour change**			

3.1 Is able to perform the change (action-efficacy)	Guided action planning [53].	Subskill demonstration, instruction, and enactment with feedback	People answer questions (from a graphic organiser, figure 2) on how they will make the change and which preparation is necessary (such as shopping). This is presented as their action plan.

3.2 Makes the change at the chosen moment (cues to action)	Learn to use cues by implementation intentions (II) [19].	Existing positive intentions and clear cues for action	Guided setting of implementation intentions for initiation of action. They state where when and how the change will be made.

3.3 Receives support from others when necessary (social support)	Mobilise social support	Combines caring, trust, openness, and acceptance with support for behavioural change.	People are motivated for and guided in asking significant others to support their behaviour change. They can talk with other participants on the forum of the intervention website.

**PO 4. People change their DI or PA**			

4.1 Is able to monitor behavioural change and compare it with goal (awareness)	Personal feedback and prompt self-monitoring	Feedback that is individual, follows the desired behaviour closely in time.	People answer questions about their behaviour change over the past week. Next, tailored feedback about performance is given.

4.2 Feels able to pick up change after lapse (maintenance-efficacy)	Reattribution training to prevent relapse [20,54].	Requires counselling of unstable and external attributions for failure.	People are asked to describe the situation that caused failure. Feedback: concentrate on the success. Learns that a lapse is normal, and that one can learn from it. It is explained to them that the situation caused the failure, but that failure can be prevented by preparing for this situation.

4.3 Identifies high-risk situations (awareness) Has possible coping strategies available. (self-efficacy?)	Relapse prevention Planning coping responses Implementation intentions [20,40])	Identification of high-risk situations and practice of coping response.	After describing the failure situation, people receive tailored advice on how to act in this specific situation (cognitive and behavioural). The coping response is formulated as an implementation intention: 'If difficult situation X arises, I'll do Y'

**PO 5. People evaluate the success of the behaviour change and its effect on body weight**.			

5.1 Is able to monitor (changes in) body weight (awareness) Is aware of normal weight range (awareness of standards)	Monitoring Guided practice [55] Visualisation of personal feedback.	Subskill demonstration, instruction, and enactment with feedback Familiar physical or verbal images as analogies to a less familiar process.	It is briefly explained why weight monitoring is done and how it should be done. At the same time, guided practice is applied to learn the steps of evaluating body weight in practice. People fill out their bodyweight every week. After four weeks, the program provides them with information about the 'normal range' of their bodyweight, and what it means if they cross this range. Visuals are used to make this visible.

5.2 Attributes weight changes correctly Shows confidence in WGP (maintenance self-efficacy)	Guided pratice Reattribution training	Requires counselling of unstable and external attributions for failure.	People passively learn how to recognise and attribute weight gain, and the actions to be taken when weight gain is observed [20].

5.3 Shows commitment to WGP (attitude/commitment)	Behavioural contract [25]	Should include goal, timeline and rewards, respondent has to agree.	People are asked to sign a personalised 'certificate', which includes tailored information from previous parts of the intervention.

**Other components of the website:**			

Receives support from others when necessary (social support)	Plan social support	Combines caring, trust, openness, and acceptance with support for behavioural change.	The GRIPP website also includes a forum, to stimulate interaction with other participants.

Knows and can cook healthy dishes	Active learning	Time, information and skills	The website includes a database with healthy recipes from all food groups.
	
	Prompt cues	Existing positive intentions and clear cues for action	A selection of useful websites is presented. This includes website on prevention of PA injuries, healthy recipes, etc.
			
Knows where to find other information about healthy food and exercising			

### Step 3 Theory-Based Methods and Practical strategies

In step 3 we identified and selected theoretical methods for modifying the important determinants and thus achieving the COs. These are to be translated into practical strategies. For each determinant we retrieved potentially applicable methods, strategies and their parameters for use (i.e. consideration to ensure effectiveness) from the theoretical and empirical literature. We then selected methods for inclusion in the intervention on the basis of technical options, feasibility, parameters for use and strategies identified in the FGIs, so that we could include one or two methods per change objective. The most important methods and strategies are shown in table 2. We will now provide some more detailed examples of methods and strategies that were chosen and applied in the program.

#### Personalised risk information

**Figure 1:**
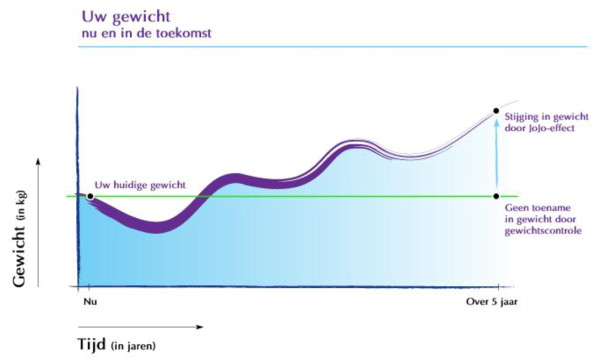
**Example:visualisation of past weight gain**. Title of the graph: 'Your weight: now and in the future. On the y-axis: 'bodyweight in kg', on the x-axis: 'time in years' (from present until 5 years in the future). The black dot on the left represents: 'your weight at present', and the upper black dot on the right represents: 'your weight in 5 years, increased because of weight cycling'. The lower black dot represents 'your possible weight when weight gain is prevented'.

Personalised (visual) risk information [23] was, for example, selected as a suitable method for increasing the perceived risk of weight gain (PO 1). With regard to the parameters for feasibility and use (individualisation, and the plausibility of the scenario), tailored graphs were shown depicting the development of expected body weight over the next five years, based on a person's weight history in the past five years (figure 1) and their future weight goal. People who had indicated WGP, substantial weight loss, or doing nothing as their future weight goal were provided with respectively a graph depicting a flat line (representing weight maintenance), a cycling curve that gradually goes up (representing weigh cycling), or a straight line that gradually goes up (representing increase in weight). All graphs were accompanied by a text explaining how it should be interpreted.

#### Decisional balance

To establish a positive attitude towards WGP (PO 1) the users were asked to review their beliefs about WGP. Their perspectives were taken into account by asking him/her what the perceived advantages and disadvantages of WGP are. The answers were presented in a decisional balance format. Each user was asked to consider both advantages and disadvantages, then to determine which are more important and last, to decide whether he/she was willing to prevent weight gain.

#### Behavioural feedback

Behavioural feedback on DI and PA was identified as a suitable method for increasing awareness of the current DI and PA (PO 2) [18,24] and to identify areas of change. By taking account of the parameters for use (individualized and specific feedback that follows the behaviour closely in time), this intervention component assesses behaviour over the past month, provides feedback on the behaviour and indicates in which specific DI or PA sub-behaviours changes of 100 kcal a day would be feasible.

On the basis of their own preference and feasibility of making small changes in DI or PA, users can choose the change that they want to proceed with. Users that prefer to make a change in DI, can start with completing the 112-item DI-questionnaire (DI-q), which assesses frequency, quantity and type (high energy - low energy) of dairy products, bread spreads, carbohydrates, meat, gravy and sauces, sugar-sweetened drinks, snacks and alcohol usually consumed during a day. After completing the questionnaire, users receive feedback on personal energy intake for each food category, illustrated by green, orange or red scores. Participants can click on each food category, to receive more detailed feedback on why they had a certain score (red or orange indicating that it would be possible to make a small change in that food category) and suggestions for what they can change in order to reduce energy intake with 100 kcal a day (see additional File 1 for an example). For PA, a thirty-five item questionnaire is used to analyse total daily PA and PA in four specific sub-domains (PA in leisure time, at work, for transportation and sports). Orange and red scores indicate the sub-behaviours in which improvements can be made. From the feedback provided, people can choose what they would like to change.

#### Goal setting

**Figure 2:**
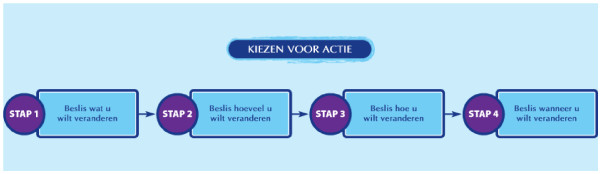
**Graphic organiser indicating the steps that need to be taken to plan for action**. Text in figure: Deciding for action (title). Step 1: Decide what you would like to change. Step 2: Decide how much you would like to change. Step 3: Decide how you would like to make the change. Step 4: Decide when you would like to make the change.

Goal setting and formation of implementation intentions were identified as appropriate strategies for preparing a behavioural change (PO 3). Important parameters for goal setting are that a user shows commitment to the goal and that goals are challenging but achievable within the individuals' possibilities. To ensure their commitment, users choose a goal that best matches their preferences and abilities. The program guides participants in defining a goal, by asking them in which DI or PA sub-domain, as indicated in the feedback (e.g., snacks), they want to make a change, to specify more exactly what they would want to change in that category (e.g., cut down on eating peanuts), how much they would like to change and whether they want to completely omit the product, or replace it by a low energy alternative (e.g., a rice cracker). Finally, users are asked to indicate when they would like to change, starting the sentence with 'If:.' (e.g. If I watch television in the evening). All choices could be written down in text boxes within the program. To support correct answers, example answers were given for every question. The answers to these four steps are summarised in a 'if... then...' statement (e.g., 'If I'm watching television in the evening, then I will eat a rice cracker instead of 2 handfuls of peanuts'). Users are advised to read the plan thoroughly and print it. A graphical representation of this process is shown in figure 2.

#### Coping planning

To facilitate the phase of goal-striving behaviour (PO 4), coping planning was identified as a suitable method to prepare people for high-risk situations. Prerequisites for this method are the identification of high-risk situations and practice of coping response, which requires instruction and demonstration. Users are instructed and guided in the action-planning process. This is applied in the second and third visit, after users have initial experience with the execution of their plans. They are asked to think of a situation that took place during the past week in which they were unable to meet their goal and choose this situation from a list (or describe the situation when it was not listed). Then, suggestions are given for strategies to cope with the chosen situation. Subsequently, users are asked to write down in pre-determined boxes what they will do the next time the difficult situation arises. Next, they are asked to think about this situation and imagine themselves performing the coping plan as a practice for the actual situation.

#### Contracting

Weight-management requires long-term commitment (PO 5). Contracting may help to remind people of their commitment and may be useful for long-term change because it increases the likelihood of self-modification by inducing self-monitoring. Important parts of a behavioural contract [25] are the statement of a clear goal, as well as a timeline and a reward plan.

The information on self-regulation of body-weight and behaviour from previous modules is summarised in a 'certificate of enrolment'. The certificate is personalised, states that person X has finished the program, and provides a personalised overview of the behavioural goals set during the program, outlining the steps of self-regulation of behaviour and bodyweight. Participants are asked to sign the certificate and print it.

#### Combining strategies

The above specified methods and strategies represent a selection of the methods and strategies used in the intervention program. The other methods were similarly translated into practical strategies that were subsequently developed into intervention components. Columns 2-4 of table 2 show the methods, parameters for use and strategies for a selection of the change objectives.

Computer-tailoring is a technique that enables the provision of individualized and personally relevant feedback and information to large numbers of people. Therefore, it was decided that the intervention would be developed as a computer-tailored program and be provided over the Internet. Computer-tailoring has proven to be a suitable method for initiating and maintaining changes in DI [26,27] and PA behaviours [28,29] and is also thought to be useful for interventions to promote weight-management [13,30,31]. The chosen theoretical methods and strategies were embedded within the tailored program.

### Step 4 Creating a coherent program

In this step, the strategies were combined within one program plan that led to the development of the actual program. Below, we outline the scope and sequence of the final program.

#### Scope, delivery

**Figure 3:**
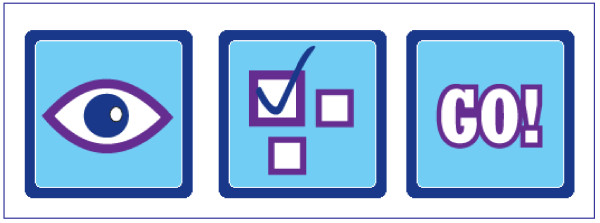
**Icons for Watch, Decide and Act**. n.a.

The main theme of the program is 'controlling your body weight'. The program was developed around the main steps of self-regulation: monitoring body weight and behaviour ('Watch'), establishing and setting goals ('Decide'), and planning and actively pursuing them ('Act'). Throughout the program these steps were symbolized by icons (see figure 3). The final program was called GRIPP, a name that was derived from getting a grip on your bodyweight, and thus to being able to control your weight.

#### Sequence

The final program consists of four parts and users are asked to visit each of the program parts during four consecutive weeks in order to work through the whole program. The first part is the most elaborate, and can take up to 45 minutes. The follow-up sessions are shorter, but the length of each visit depends on the answers of the user. In total, it takes about 90 minutes to finish the program.

**Figure 4:**
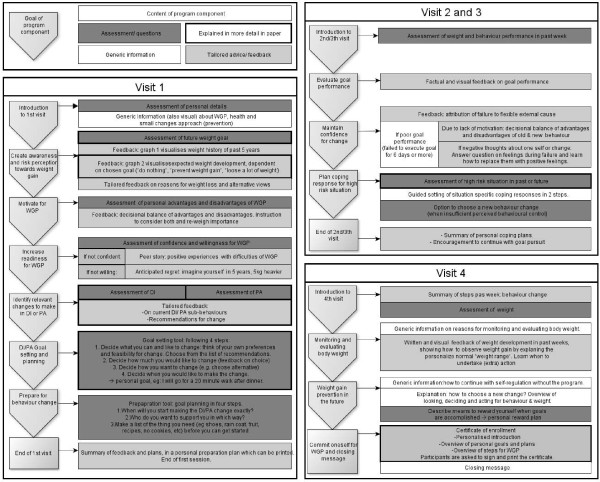
**Overview of the program**. Starting with 'Visit 1', this figures shows the main steps of the GRIPP program.

The main goals of the first visit are to motivate people for weight gain prevention (PO 1), have them choose one behaviour change (PO 2) and plan action initiation (PO 3). The main goals of the second and third visit are to evaluate behaviour change in the past week, and to adapt action and coping plans (PO 4). The main goal of the fourth visit is to learn how to maintain self-regulation of body weight in the future, without use of the program (PO 5). The sequence is shown in figure 4.

#### Development

The tailored program was developed using Tailorbuilder (c) software, which supports the creation of an entire internet-based computer-tailored program without a programmer. The tailored program was delivered as part of a project website that also included a forum (to generate support and share experiences with other participants), a recipe database and links to useful websites.

#### Pre-test of the intervention

A prototype of the program was pre-tested in order to identify aspects of the program that could be improved. Forty-eight adults aged 25-64 years with a BMI between 25-30 kg/m^2 ^(i.e. representatives of the target group) participated in the pre-test.

The most important findings of the pre-test were that the participants liked and appreciated the program, particularly the interactive elements. All information was understood well. The program was also considered to be long, not always clear in structure and navigation, and the follow-up sessions were perceived as being less tailored than the first session. On the basis of these findings, the program was considerably reduced in length by reducing the amount of text per page and the number of pages. Changes were made to improve ease of navigation and attractiveness. The content of follow-up sessions was also made more tailored and personalised.

### Step 5 Anticipation of Adoption and Implementation

The purpose of step 5 of the IM protocol was to anticipate the adoption and implementation of the intervention. To do so, representatives from potential implementing organizations and of the target group were involved in the intervention-development process.

Representatives of potential implementing organizations participated in expert groups, which also included other researchers and intervention developers. These expert groups supported development, evaluation and implementation of the intervention. To facilitate its future implementation as an Erasmus MC program, it was designed according to the style guide of the University Medical Centre.

Representatives of the target group participated in focus-group interviews, which facilitated the identification of important determinants, important goals and potentially useable and well appreciated intervention components. By participating in the pre-tests, representatives of the target group helped to modify and improve the program.

### Step 6 Evaluation Plan

The final step of the IM process was the development of an evaluation plan, of which a brief overview is described below. The Medical Ethics Committee of Erasmus Medical Center issued a declaration of no objection for the study. The trial was registered in the Dutch Trial registry (nr 1862).

#### Design and procedure

A two-group randomized controlled trial design will be used to study the effects of the intervention by comparing the intervention with a control group that will receive generic information about weight-management. The graphic designs of the intervention and control website are identical.

Measurements will be at baseline and one-month and six-months post-intervention. Stratified block randomisation to either the intervention or control group will take place after completion of the baseline measures. Participants will have access to their assigned study website for 2 months. To prompt website use bi-weekly e-mail reminders will be sent.

#### Participants

A total of 600 adults will be recruited for participation in the study. The required number of participants is based on a power calculation, in which 400 participants would be sufficient to detect an intervention effect of 0.4 BMI points with a power of 0.80 and a significance level of p < .05. To account for drop-out between the measurements we will recruit 600 participants for the study.

The main inclusion criteria for participation are being overweight (BMI = 25-30 kg/m^2^) and aged between 25 and 65 years. In addition, sufficient command of the Dutch language and access to the Internet is required. Exclusion criteria are a diet prescribed by a doctor or dietician, pregnancy, being physically unable to increase PA and unwillingness to participate in all parts of the study.

Participants will be recruited from the general population through advertisements in local newspapers, flyers will be delivered door-to-door and in waiting rooms of GP's, and among the employees of four large companies, with the aim to reach a diverse population with respect to socioeconomic status.

#### Program outcomes and measurements

The various levels of program outcomes that will be evaluated are determined by the goals established in the IM procedure. Because the primary objective of this study is to prevent weight gain, body weight, waist circumference and skin-fold thickness [32] will be evaluated at six months post-intervention. The measurements will be performed by trained research-assistants. Written informed consent will be obtained at baseline measurements.

Weight gain can be prevented by small changes in energy intake and energy expenditure and these behaviours are therefore secondary outcomes of this study. They are measured by self-reported, online questionnaires at baseline, one-month and six-month post-intervention. We hypothesised that self-regulation skills and other determinants of behaviour (such as attitude and self-efficacy) are mediators of DI and PA, as such they are also assessed in these questionnaires.

Other outcomes are measures of development and implementation, also referred to as the process evaluation [11]. Participants will fill out a process evaluation questionnaire at one-month follow-up, which includes questions on perceived personal relevance, appreciation, readability, difficulty and usability. The tailoring software allows for the collection of objective data about the use of the program.

**Figure 5:**
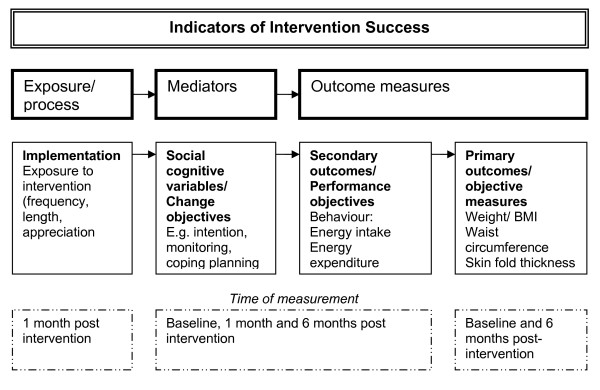
**Indicators of success and time of measurements**. n.a.

An overview of the outcomes is shown in figure 5.

## Discussion

This paper describes the systematic development of an online computer-tailored intervention intended to prevent weight gain in overweight adults. The intervention follows users over a period of three weeks and provides them with feedback at several points in time in order to promote self-regulation of behaviour and weight. It has an interactive, individualised approach and can be used by a large group of people.

The IM protocol helped us integrating insights from various theories. The performance objectives and methods were guided by self-regulation theory but empirical evidence with regard to the effectiveness of theoretical methods is limited [12]. Sometimes, feasibility issues made it necessary to deviate from the original, theory-based plans. Although IM was helpful in specifying sub-goals from general goals, it does not take into account the complexity of (sub-)goals that may need to be targeted simultaneously (e.g. DI *and *PA). These simultaneous goals generally lead to multiple mini-interventions. The tailored approach allowed us to realise that and ensured that the intervention was personalised in content as well as strategies.

The aim of this paper was to provide transparency with regard to intervention development, as suggested by Abraham and Michie [12]. We aimed to increase insight into the effectiveness of interventions and the techniques that enhance effectiveness. The design and measures of the evaluation study are based on previous steps of IM and therefore allow us to study the contribution of specific components to the efficacy. If the intervention is efficacious it may help to prevent weight gain at the population level, at relatively low costs.

## Extra

The complete tables of change objectives can be obtained through the authors on request.

## Competing interests

The authors declare that they have no competing interests.

## Authors' contributions

AO had the original idea for the study and its design. AO and PE supervise(d) the development and study. LG and IF developed the intervention. LG coordinates the study. LG drafted the manuscript, PE, IF and AO were involved in revising it. All authors read and approved the final manuscript.

## Pre-publication history

The pre-publication history for this paper can be accessed here:

http://www.biomedcentral.com/1471-2458/10/649/prepub

## Supplementary Material

Additional file 1 **Example of personal feedback on dietary intake**. n.a. Click here for file
