# Supplementary material for: Systematic development of a self-regulation weight-management intervention for overweight adults
Source: BMC Public Health. 2010 Oct 27;10:649. doi: 10.1186/1471-2458-10-649 (PMC2988733; doi:10.1186/1471-2458-10-649)
Supplement: Additional file 1 — Example of personal feedback on dietary intake. n.a. [file 1471-2458-10-649-S1.DOC]

Additional file 1. Example of personal feedback on dairy intake.

*On a daily basis, you drink and eat the following dairy products:*

*3 glasses of full fat milk*

*1 cup of sweetened porridge*

On average, you eat more than 3 portions of dairy per day. According to the dietary guidelines, 400 ml or 3 portions is sufficient for a woman of your age.

You can reduce your energy intake from dairy products by reducing the amount of dairy products. You can also choose to replace some of the energy-dense products you are currently eating by products that are less energy dense.
